# Engineering lanthipeptides by introducing a large variety of RiPP modifications to obtain new-to-nature bioactive peptides

**DOI:** 10.1093/femsre/fuad017

**Published:** 2023-04-24

**Authors:** Yuxin Fu, Yanli Xu, Fleur Ruijne, Oscar P Kuipers

**Affiliations:** Department of Molecular Genetics, Groningen Biomolecular Sciences and Biotechnology Institute, University of Groningen, Groningen 9747 AG, The Netherlands; Department of Molecular Genetics, Groningen Biomolecular Sciences and Biotechnology Institute, University of Groningen, Groningen 9747 AG, The Netherlands; Department of Molecular Genetics, Groningen Biomolecular Sciences and Biotechnology Institute, University of Groningen, Groningen 9747 AG, The Netherlands; Department of Molecular Genetics, Groningen Biomolecular Sciences and Biotechnology Institute, University of Groningen, Groningen 9747 AG, The Netherlands

**Keywords:** lanthipeptides, post-translational modification, ripps, Engineering, NRPs mimics, synthetic biology

## Abstract

Natural bioactive peptide discovery is a challenging and time-consuming process. However, advances in synthetic biology are providing promising new avenues in peptide engineering that allow for the design and production of a large variety of new-to-nature peptides with enhanced or new bioactivities, using known peptides as templates. Lanthipeptides are ribosomally synthesized and post-translationally modified peptides (RiPPs). The modularity of post-translational modification (PTM) enzymes and ribosomal biosynthesis inherent to lanthipeptides enables their engineering and screening in a high-throughput manner. The field of RiPPs research is rapidly evolving, with many novel PTMs and their associated modification enzymes being identified and characterized. The modularity presented by these diverse and promiscuous modification enzymes has made them promising tools for further *in vivo* engineering of lanthipeptides, allowing for the diversification of their structures and activities. In this review, we explore the diverse modifications occurring in RiPPs and discuss the potential applications and feasibility of combining various modification enzymes for lanthipeptide engineering. We highlight the prospect of lanthipeptide- and RiPP-engineering to produce and screen novel peptides, including mimics of potent non-ribosomally produced antimicrobial peptides (NRPs) such as daptomycin, vancomycin, and teixobactin, which offer high therapeutic potential.

## Teaser

Potent non-ribosomal antimicrobial peptides can be mimicked by in vivo combining various posttranslational modifications of gene-encoded lanthipeptides, allowing high throughput screening of large mutant libraries.

## Introduction

**Figure 1.**
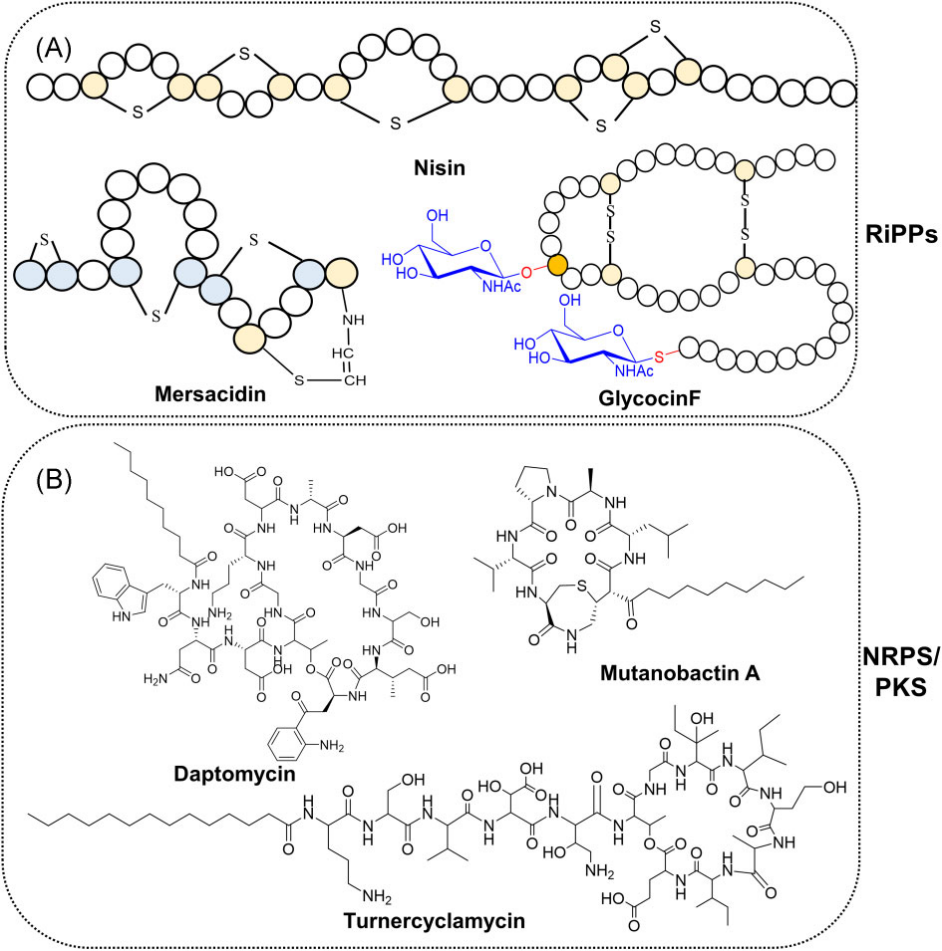
Structural representatives of diverse NRPs and RiPPs. (A) Examples of RiPPs from Lactic Acid Bacteria (LAB). Mersacidin is ribosomally produced by *Bacillus* sp. strain HIL Y-85, 54 728; Glycocin F (GccF) is a potent bacteriocin originally isolated from liquid culture of *Lactobacillus plantarum* KW30; and Nisin is a penta-cyclic antibacterial peptide produced by the bacterium *L. lactis*. (B) Examples of NRPs/PKS. Daptomycin is a cyclic lipopeptide antibiotic produced by *Streptomyces roseosporus* i.e. used for the treatment of serious Gram-positive infections; Mutanobactin A is a hybrid PKS-NRPS isolated from *Streptococcus*; and Turnercyclamycin, produced by *Teredinibacter turnerae*, is a lipopeptide antibiotic against several Gram-negative pathogens.

Lanthipeptides represent a major group of ribosomally encoded and post-translationally modified peptides (RiPPs), produced by a large variety of microorganisms (Arnison et al. 2013a; Montalban et al.^2021^)), including various strains of lactic acid bacteria (LAB), such as *Lactococcus lactis*, which produces the paradigm lantibiotic nisin (Fig. 1A) (Rogers and Whittier 1928, Gross and Morell 1971, Kuipers et al. 1993, 1995; Lubelski et al. 2008, de Arauz et al. 2009, Cooper et al. 2010). Lanthipeptides are synthesized from a genetically encoded precursor peptide, LanA, which consists of an N-terminal leader region involved in the recognition of the biosynthetic machinery and a C-terminal core region that undergoes post-translational modifications (PTMs). Lanthionine (Lan) and/or methyllanthionine (MeLan) rings are the most characterized PTMs in lanthipeptides, which are introduced in a two-step process. In the first step, Ser and Thr residues in the core peptide of LanA are dehydrated to dehydroalanine (Dha) and dehydrobutyrine (Dhb) residues, respectively, by a dehydratase. Lan/MeLans are subsequently formed via Michael-type addition of sulphydryl groups of Cys residues onto the Cβ−atom of dehydroamino acids. The resulting cyclic peptides have constrained conformations that confer stability and assist in various biological activities. Lanthipeptides can be divided into five classes based on the biosynthetic enzymes that produce them; the synthetases of these classes have been discussed in previous studies (Zhang et al. 2012, Repka et al. 2017, Kloosterman et al. 2020, Pei et al. 2022).

In recent years, the rapidly expanding field of RiPPs investigations has revealed a vast range of PTMs that confer important biological properties to these molecules. As of 2021, 41 known classes of RiPPs have been defined, each class defined by its corresponding PTM (Montalbán-López et al. 2021). The wide range of moieties installed in RiPPs, such as thiazole/oxazole heterocycles and epoxide groups, have not been reported to occur in lanthipeptides, which suggests that a vast source of PTM enzymes can offer their untapped potential for lanthipeptide engineering to yield novel exotic structures in (lanthi) peptides. These extensive enzymatic PTMs confer important biological properties to RiPPs, for instance head-to-tail cyclization and the signature cyclic cysteine knot (CCK) motif, which is essential for the antiviral activity of cyclotides (Daly et al. 2004, Fu et al. 2021). In addition, a *N,N*-dimethyl-alanine introduced by the SAM-dependent methyltransferase in the linaridin cypemycin is vital for its antibiotic activity (Claesen and Bibb 2010), and a Tyr-Ile ether crosslink provides important contacts for tubulin-binding and thereby potent antimitotic activity of phomopsins (Morisaki et al. 1998, Cormier et al. 2008). RiPP biosynthetic enzymes thus offer a valuable biocatalytic toolbox to install diverse chemical structures into peptides, thereby conferring novel bioactivities. Combinatorial application of these tools in lanthipeptide biosynthetic assembly lines would allow for the generation of new-to-nature.

**Figure 2.**
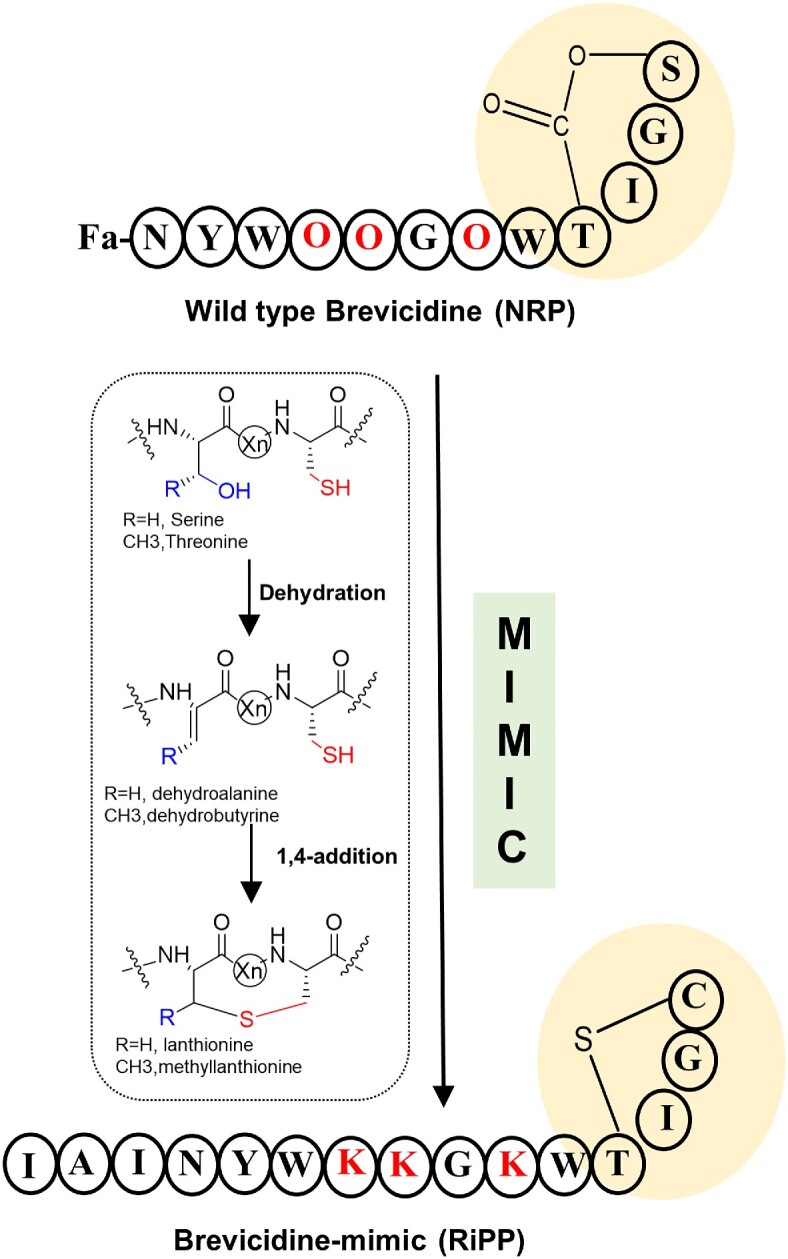
Schematic diagram of the non-ribosomal peptide Brevicidine mimicked by ribosomal synthesis. NRPs are non-ribosomal peptides; RiPPs are ribosomally synthesized and post-translationally modified peptides.

Non-ribosomal peptides (NRPs) (e.g. the antibiotics daptomycin, vancomycin, and teixobactin) represent another class of peptide secondary metabolites (Fig. 1B), with enormous structural and functional diversity as well as high therapeutic potential, which has triggered interest in their potential engineering to develop improved antibiotic variants (Felnagle et al. 2008, Bozhüyük et al. 2018, 2019; Liu et al. 2019, Huang et al. 2021, Wenski et al. 2022). However, unlike the biosynthetic plasticity and adaptability of genetically encoded peptides (Montalbán-López et al. 2017, Wu and van der Donk 2021), NRPs are synthesized by large biosynthetic complexes that are difficult to functionally express and engineer (Felnagle et al. 2008, Kries 2016, Süssmuth and Mainz 2017). To overcome the challenges of NRPS re-engineering to generate improved NRP variants, a strategy has been proposed to use lanthipeptides as starting points to synthesize peptides with similar NRP structural features by employing RiPP biosynthetic pathways. (Van Der Velden et al. 2017, Mordhorst et al. 2020, 2023, Zhao et al. 2020, Ruijne and Kuipers 2021). As shown in Fig. 2, the molecular structure of the antimicrobial NRP brevicidine could be partially mimicked by ribosomal synthesis, introducing a cyclic structure by Melan ring formation by the lanthipeptide synthetase involved in the biosynthesis of nisin, NisBC. The resulting engineered lanthipeptide displayed a similar antimicrobial activity and mode of action as the wildtype NRP brevicidine, demonstrating the feasibility of this strategy (Zhao et al. 2020).

However, the construction of biosynthetic assembly lines for the sustainable production of novel lanthipeptides with diverse structures is limited by a number of non-negligible factors. The substrate specificity of the enzymes that generate the tailoring PTM is one of them, as exemplified by NisBC and the Lan/MeLan ring-installation enzymes involved in nisin biosynthesis. The dehydration activity of Ser/Thr residues of dehydratase NisB depends in part on the amino acids flanking the dehydratable; Ser/Thr residues, e.g. a negatively charged Asp residue preceding a serine disfavours its dehydration by wild-type NisB (Zhao et al. 2020), whereas the cyclase NisC is not capable of catalysing the formation of exceptionally large rings in peptide substrates (unpublished result). In view of this, only a limited number of substrates are suitable for NisBC-mediated Lan/MeLan ring installation. The leader-dependent mechanism to direct some lanthipeptide PTM enzymes to C-terminal substrate sequences needs to be taken into account when applying these enzymes in combinatorial biosynthesis (Plat et al. 2011, Montalbán-López et al. 2017). The leader peptides govern the post-translational tailoring processes on specific substrates, eliminating undesired effects on the modification of other peptides/protein products and keeping modified peptides inactive within the producing cell. It may also pose a barrier to the introduction of multiple additional PTMs into one specific substrate (Lagedroste et al. 2020, Lagedroste et al. 2021). The identification of some leader-independent modification enzymes (such as the reductase LtnJ involved in lacticin 3147 biosynthesis, Cotter et al. 2005) and the development of strategies for a combination of different PTM enzymes in one assembly line, such as the ‘hybrid leader’ strategy, based on the observations that only a limited region of the leader peptide in certain RiPPs is required for efficient modification of the peptides (Burkhart et al. 2017), provide possible solutions to this concern. Thus, this calls for the discovery and implementation of more PTM-installation enzymes to form a candidate pool that can be selected from and combined into biosynthetic assembly lines to generate lanthipeptide libraries. Besides dehydration and Lan/MeLan ring formation, an extended range of further modifications such as D-amino acid incorporation (Ryan et al. 1999, Cotter et al. 2005), halogenation (Castiglione et al. 2008, Foulston and Bibb 2010), methylation (Grigoreva et al. 2021), hydroxylation (Zimmermann et al. 1993, Huo et al. 2017), and acylation (Ozaki et al. 2017) have been observed in lanthipeptides. Although the number of reported PTMs and associated enzymes in lanthipeptides is still limited, an expanded repertoire of PTMs can be found in other RiPPs classes that offer an extensive additional array of structural moieties and bioactivities.

In this review, we explore recent insights into PTM biosynthetic enzymes from RiPPs, emphasizing their application as catalytic tools in lanthipeptide engineering. We also discuss the combinatorial biosynthesis strategies for efficient diversification of lanthipeptides, including the possibility of structurally and functionally mimic NRPs. High-throughput methods for screening bioactive peptides with desired therapeutic properties will also be addressed.

## RiPP biosynthetic enzymes with engineering potential

PTMs involve enzyme-mediated addition of a wide range of moieties in RiPPs at their amino acid side chains, such as cyclization, hydroxylation, halogenation, and acylation, and can also occur at peptide linkages or at the N- or C-terminus, as in epimerization, β-amino acid incorporation, and methylation (for several of these structures, see Table 1). These PTMs provide RiPPs with diverse structures that regulate their binding and affinity to biological targets and confer promising scaffolds for pharmaceutical applications. Many PTM-associated enzymes have been identified by genome mining across all kingdoms of life and have been produced and characterized using homologous or heterologous expression systems, which have allowed for a deep biochemical understanding of their catalysis and thereby paved the way for using RiPP maturases in synthetic biology. The enzymes, especially those with exceptional substrate tolerance, allow for the creation of a versatile catalytic toolbox that can be used for lanthipeptide and other RiPPs engineering, ultimately creating novel peptide drugs with desired properties. In this chapter, we discuss various RiPP enzymes that install a diverse set of PTMs and highlight examples and prospects for their engineering application in lanthipeptides.

**Table 1.** Examples of various possible modifications and therepresentative enzymes of their respective RiPPs.

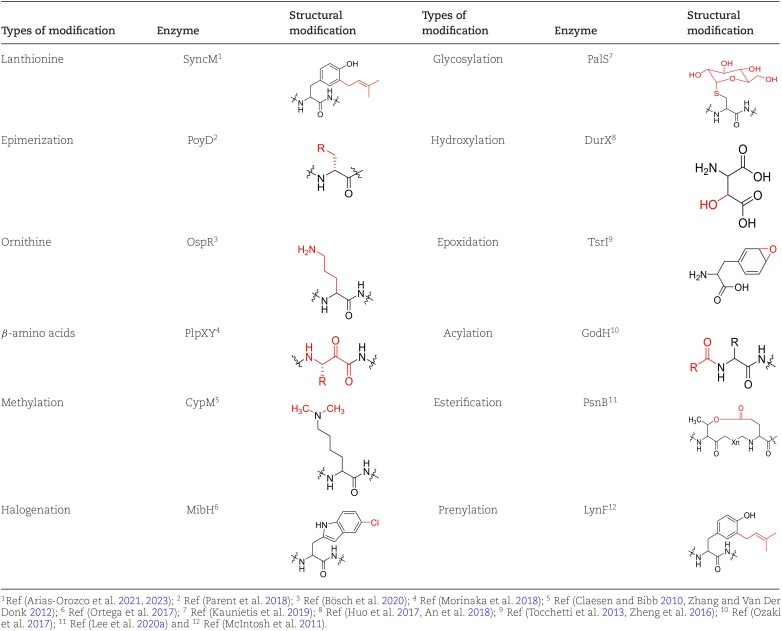

### Dehydration and cyclization

Dehydration is a frequently observed modification across various RiPP families, such as lanthipeptides, linaridins, cyanobactins, and thiopeptides (Ortega and Van Der Donk 2016, Mo et al. 2017, Ma and Zhang 2020, Vinogradov and Suga 2020). Dehydro amino acids often act as an initiation step for further PTMs, as exemplified by lanthipeptides, where Ser and Thr residues in the core peptide of LanA are dehydrated to Dha and/or Dhb residues by a dehydratase domain. These dehydroamino acids can be involved in subsequent cyclization to form Lan/MeLan rings and can also be used as substrates for subsequent hydrogenation to generate D-Ala and D-Abu. In addition, dehydroamino acids themselves also provide functionality to peptides. For example, substituting Dha5 for alanine in the lantibiotic subtilin had no effect on its action against vegetative *Bacillus cereus* T cells but abolished its inhibition of spore outgrowth (Liu and Hansen 1992, 1993). In addition, the nisin mutant Dha5Ala was shown to have activity very similar to that of wild-type nisin in inhibiting the growth of *L. lactis* and *Micrococcus luteus*, but was significantly less active than nisin as an inhibitor of the outgrowth of spores of *Bacillus subtilis* (Chan et al. 1996). Furthermore, a nisin-inactivating enzyme, i.e. present in several nisin-resistant strains, was identified as a Dha reductase, which inactivates nisin as well as subtilin by reducing a C-terminally located Dha to Ala (Jarvis 1967, Jarvis and Farr 1971). Overall, these observations suggest the important role of dehydro amino acids in the activity and mechanisms of action of certain lanthipeptides, which also offers the basis for the introduction of other PTMs.

Cyclization is a widespread type of peptide modification that constrains the conformation of peptides, providing proteolytic stability, rigidity, and bioactivity (Funk and Van Der Donk 2017). The impact of cyclization on membrane permeability, bioavailability, and pharmacokinetic properties has also been studied (Driggers et al. 2008, Naylor et al. 2017, Price et al. 2017). Cyclic RiPPs are generated by ribosomal synthesis of a linear peptide, i.e. subsequently modified with heterocyclic and/or macrocyclic structures by a single enzyme or group of enzymes. To date, various enzymatic RiPP macrocyclization reactions have been revealed, including head-to-tail ligation, lanthionine ring formation, AviCys/AviMeCys ring formation, radical S-adenosylmethionine (SAM)-dependent enzyme-catalysed ring formation, and other types of ring formation brought about by intramolecular crosslinks. Several enzymes that catalyse cyclization have been experimentally characterized and successfully applied as synthetic biology tools in peptide engineering, as will be discussed in this section.

Lanthionine (Lan) and/or methyllanthionine (MeLan) rings are characteristic structural features of lanthipeptides. The formation of Lan/MeLan motifs in class I lanthipeptides is catalysed by two different enzymes, a lanthipeptide dehydratase (LanB) and a lanthipeptide cyclase (LanC), which are exemplified by NisB and NisC from the nisin biosynthetic gene cluster. NisBC enzymes are well established for their use in lanthipeptide engineering, as has been shown in many examples. This includes the installation of a lanthionine ring in vasopressin (a neurohypophysial hormone) (Li et al. 2019), as well as the production and modification of many new-to-nature lanthipeptides with potent antimicrobial activity (Van Heel et al. 2016). For Class II lanthipeptides, a bifunctional lanthipeptide synthetase, LanM, catalyses both dehydration and cyclization of precursor peptides (Table 1). SyncM from the marine cyanobacteria *Synechococcus* MITS9509 is the most promiscuous lanthipeptide synthetase described to date. The very relaxed substrate specificity of SyncM towards its precursors and the ability to catalyse the formation of exceptionally large rings in a variety of topologies suggest that SyncM could be an attractive tool to design and produce a variety of new-to-nature lanthipeptides with a broad range of ring topologies (Arias-Orozco et al. 2021, 2023). Besides Lan/MeLan rings, the lysinoalanine (Lal) crosslink forms another macrocyclic structure, e.g. found in the lanthipeptide duramycin. The DurN protein from the duramycin BGC is proposed to generate LaI formation by catalysing the addition of the C-terminal Lys to a Dha residue in the peptide in a substrate-assisted, leader peptide-independent way (An et al. 2018).

The C-terminal 2-aminovinyl-cysteine/2-aminovinyl-3-methylcysteine (AviCys/AviMeCys) macrocyclizations are unique structures found in many lanthipeptides (e.g. in epidermin and mersacidin) and also exist in other RiPPs families, including linaridins (e.g. cypemycin), lipolanthines (e.g. microvionine), and thioamitides (e.g. thioviridamide) (Hayakawa et al. 2006, Claesen and Bibb 2010, Mo et al. 2019). The current model for the biosynthesis of AviCys/AviMeCys involves a multi-step enzymatic process that requires the co-mediation of a dehydratase, cyclase, and flavin-dependent Cys decarboxylase (generically termed LanD in lanthipeptide biosynthesis). The decarboxylase LanD is well characterized in several examples, including MrsD from mersacidin biosynthesis and CypD from cypemycin biosynthesis (Ding et al. 2018, Mo et al. 2019, Viel et al. 2021). Moreover, recent investigation of the enzymes involved in AviCys formation, such as MicKC-MicD in microvionin biosynthesis and LxmKXYD for lexapeptide, led to the discovery of the multi-enzyme cooperative biosynthetic strategy for this type of macrocyclization (Wiebach et al. 2018, Xu et al. 2020, Lu et al. 2021b).

Examples of head-to-tail cyclized RiPPs include large antimicrobial peptides (*e.g*. enterocin AS-48, circularin A), cyanobactins from cyanobacteria (*e.g*. patellamides and trunkamides), plant-derived cyclotides (e.g. kalata B1), orbitides (e.g. cyclolinopeptide A), and fungal peptides (e.g. omphalotin A, α-amanitin and phalloidin) (Mayer et al. 1997, Bell et al. 2000, Kawai et al. 2005, Burgos et al. 2014, Weidmann and Craik 2016). The cyanobactin macrocylase PatGmac is a subtilisin-like protease involved in head-to-tail cyclization of patellamide. It recognizes the C-terminal macrocyclization signature (positions P1′–P4′) of AYDG and requires a proline residue at the P1 position before the cleavage site. In unmodified peptides, the C-terminal macrocyclization signature is cleaved off to form an acyl-enzyme intermediate, i.e. subsequently converted to a peptide bond (Koehnke et al. 2012). PatGmac allows several changes in the substrate sequence, and this substrate tolerance has been successfully used to produce a broad range of macrocyclic peptides *in vitro* (Houssen et al. 2014). Another macrocyclase, OscGmac, encoded within the cyanobactin oscillacyclamide A and B gene cluster, was reported to have an even higher substrate tolerance than PatGmac and can process substrates without the conserved proline/thiazoline at position P1. Furthermore, OscGmac can cyclize peptides that are longer than PatGmac substrates, including peptides containing D-amino acids (Alexandru-Crivac et al. 2017). OscGmac thus offers a useful biotechnological tool in peptide engineering to install head-to-tail macrocyclization in a variety of peptides.

Unlike head-to-tail cyclizations formed by attack of the N-terminal amine of the core peptide onto the C-terminus, side-chain macrolactam/macrolactone are cyclizations formed between the N-terminal amine of the core peptide and a side-chain carboxylate or between a side-chain amine and a carbonyl, which often occur in the biosynthesis of lasso peptides (e.g. microcin J25 and fusilassin) (Rosengren et al. 2003, Dicaprio et al. 2019) and microviridins (e.g. microviridin B) (Amaral et al. 2021). A recent *in vitro* study showed that FusC (an ATP-dependent lasso cyclase i.e. homologous to asparagine synthetase) from the fusilassin pathway is capable of producing millions of sequence-diverse lasso peptides and displays a large substrate tolerance, showing great potential for synthetic biology applications (Si et al. 2021).

A subfamily of RiPPs containing distinct intramolecular ω-ester or ω-amide bonds that connect the carboxyl side chain of glutamate or aspartate with a hydroxyl side chain of threonine or serine or with an amine side chain of lysine, named ω-ester-containing peptides (OEPs). The first identified macro-cyclized and well-characterized OEPs are the microviridins (Ishitsuka et al. 1990). Recently, novel structures were added to this sub-family, like plesiocin, in which the ester bond is formed by linking of the side chain of amino acids threonine and glutamic acid, catalysed by PsnB (Table 1) (Molohon et al. 2011, Lee et al. 2020a). Thuringinin (Roh et al. 2019) and fuscimiditide (Elashal et al. 2022) also belong to this sub-family. Genome mining approaches also demonstrated the natural diversity of OEPs (Lee et al. 2020b, Ramesh et al. 2021). Compared with other end-to-side linkage peptides, OEPs have the obvious characteristic of side-to-side connections, which are modified by ATP-grasp enzymes, showing a great potential for introducing considerable structural diversification into small peptides. Therefore, further studies of ATP-grasp enzymes are of great significance as tools for the structural diversification of peptides in synthetic biology.

Furthermore, a wide range of macrocyclizations in RiPPs are mediated by radical S-adenosylmethionine (rSAM) enzymes through the construction of C–C and C–S linkages (Mahanta et al. 2017, Benjdia and Berteau 2021, Lu et al. 2021a). The C–C crosslinks between aromatic and aliphatic side chains of nearby residues occur, e.g. in streptide (Lys-to-Trp) (Schramma et al. 2015), pyrroloquinoline quinone (Glu-to-Tyr) (Barr et al. 2016), ryptides (Arg-to-Tyr) (Caruso et al. 2019), and triceptides (Trp/Phe-to-Asn/Lys/Gln/Arg/Asp/Ser crosslinks) (Nguyen et al. 2020). The C–S crosslinks are widespread in sactipeptides, with subtilosin A as a representative peptide, which contains two Cys–Phe crosslinks and one Cys–Thr (S–αC) crosslink (Kawulka et al. 2004, Flühe et al. 2012). In recent bioinformatic research, more RiPPs with C–S crosslinks were identified, such as freyrasin, which contains six Cys–Glu (S–βC) crosslinks, and thermocellin, which contains a Cys–Thr (S–γC) crosslink (Hudson et al. 2019). Notably, rSAM enzymes were also found to be able to install six-membered heterocycles into peptides, such as by forming C–O crosslinks between Thr–Gln in rotapeptides (Clark et al. 2019) and an α-thioether C-S bond, joining neighbouring Cys and Arg residues in enteropeptins (Clark et al. 2021).

RiPPs modified with thiazole/oxazole heterocycles derived from cysteine, serine, and threonine residues were identified in a number of different families, including linear azol(in)-containing peptides (LAPs) (Sinha Roy et al. 1998), cyanobactins (Schmidt et al. 2005), thiopeptides (Engelhardt et al. 2010), and bottromycins (Huo et al. 2012) (Melby et al. 2011). The heterocycles in natural peptides confer stability and/or electronic distributions to the peptides, thus enabling peptide–protein recognition and DNA/RNA–peptide interactions, which are thought to be important for the biological function of the modified peptide (Roy et al. 1999, Mhlongo et al. 2020, Mordhorst et al. 2023). The installation of oxazoles and thiazoles into peptides is catalysed by an enzyme complex in two steps. First, a cyclodehydratase catalyses the heterocyclization of serine/cysteine/threonine residues. Second, a flavin mononucleotide (FMN)-dependent dehydrogenase facilitates the oxidation of oxazoline/thiazoline to produce an oxazole/thiazole moiety, respectively (Gao et al. 2018, Ge et al. 2019). Several engineering studies have explored the biosynthesis potential of these cyclodehydratase–dehydrogenase pairs for the generation of oxazole-/thiazole-containing peptide analogues. For example, a hybrid leader strategy was used to direct the cyclodehydratase LynD (from aesturamide biosynthesis) and the dehydrogenase TbtE (from thiomuracin biosynthesis) to install thiazol(in)es within non-native substrates (Donia et al. 2008, Hudson et al. 2015, Fleming et al. 2019). Furthermore, a leader-peptide-free strategy can catalyse the cyclodehydration of leaderless peptide substrates containing a C-terminal Ala-Tyr-Asp recognition sequence by fusing the leader peptide to the N-terminus of the cyclodehydratase (e.g. LynD Fusion and MicD Fusion) (Oman et al. 2012, Koehnke et al. 2015, Oueis et al. 2015, Ge et al. 2019). In addition, dehydrogenases have also been reported to accept leaderless substrates (Gao et al. 2018). These findings led to the fusion of the cyclodehydratase LynD and dehydrogenase ArtGox, which are derived from different biosynthesis pathways, which allowed for the effective installation of thiazole and thiazoline heterocyclic backbones within folded proteins and diverse heteropolymers (Walker et al. 2022). This highlights the potential of these enzymes to engineer peptides with thiazole/oxazole heterocycles.

### D-amino acid incorporation

D-amino acids are non-proteinogenic residues that are present in many bioactive natural peptides, where they enhance the bioactivity and stability of bioactive natural peptides as well as confer stereochemical constraints for downstream biosynthesis into the final products (Ding et al. 2020, Mordhorst et al. 2023). While the occurrence of D-amino acids in RiPPs is relatively rare compared to their frequent presence in NRPs, a few mechanisms of D-amino acid biosynthesis in RiPP pathways have been elucidated.

A small subclass of lanthipeptides contains D-amino acids that are formed by hydrogenation of Dha and/or Dhb residues. The resulting D-Ala and D-Abu residues have been found in several lanthipeptides, such as lacticin 3147, carnolysin, and bicereucin (Ryan et al. 1999, Cotter et al. 2005, Lohans et al. 2014, Huo and Van Der Donk 2016). The enzymes that carry out the reduction reactions are encoded in these lanthipeptide gene clusters, collectively termed LanJ. These enzymes are grouped into three dehydrogenase classes (Cotter et al. 2005, Repka et al. 2017), including the zinc and NADPH-dependent dehydrogenases (e.g. LtnJ involved in lacticin 3147 biosynthesis) termed LanJ_A_, the flavin-dependent oxidoreductases (e.g. CrnJ involved in carnolysin biosynthesis) termed LanJ_B_ (Lohans et al. 2014), and the most recently discovered F_420_H_2_-dependent reductases (e.g. LxmJ involved in lexapeptide biosynthesis) termed LanJ_C_ (Xu et al. 2020).

The engineering potential of LanJ in peptide modification, especially in lanthipeptides, has been explored in several studies. For example, NpnJ_A_ has been shown to reduce dehydroalanine to D-Ala at non-native positions in a range of non-native substrates, showing flexibility for recognition with respect to the position of the dehydroalanine and high substrate tolerance in both *in vivo* and *in vitro* reconstitutions (Yang and Van Der Donk 2015). LtnJ_A_, a reductase responsible for the introduction of D-Ala in lacticin 3147, has been co-expressed with the nisin modification machinery (NisBTC) in *L. lactis* to achieve successful incorporation of D-Ala into the lanthipeptide nisin and a linear nisin variant. Notably, LtnJ_A_ does not require a leader peptide for its activity (Mu et al. 2015).

In addition, radical S-adenosylmethionine epimerases belong to an enzyme family that catalyses the regiospecific and irreversible introduction of multiple D-residues into ribosomal peptides. Two types of epimerases of this family have been reported in the literature to date (Morinaka et al. 2017). PoyD, the first type of radical SAM epimerases, installs several D-amino acids along the polytheonamide backbone as a maturation step (Table 1). PoyD, as well as several other proteusin radical SAM epimerases (e.g. OspD, AvpD, and PlpD), have been demonstrated to accept a wide range of different substrates and residue types and can introduce different epimerization patterns beyond those observed on native substrates, suggesting that these epimerases have considerable potential for peptide engineering (Morinaka et al. 2014, Vagstad et al. 2019, Korneli et al. 2021). Rational introduction of D-amino acids at desired locations is, however, challenging as the rules that govern core peptide recognition have not been elucidated to date (Morinaka et al. 2017, Korneli et al. 2021). The second type of radical SAM epimerases is represented by the epimerase YydG, which has a clearly distinct domain architecture compared to the PoyD-type of epimerases. YydG installs D-allo-Ile and D-Val into its cognate peptide substrate, YygF. YydF belongs to a novel RiPPs family named epipeptides, which was first characterized in *B. subtilis*. The bioactivity of epipeptides was demonstrated to be dependent only on epimerizations and requiring no other PTMs (Benjdia et al. 2017).

Furthermore, post-translational epimerizations have been reported in other RiPPs families as well. A recently discovered novel epimerase, MslH, installs the C-terminal D-Tyr into the lasso peptide MS-271 in a metal- and cofactor-independent manner and exhibits broad substrate specificity towards the *N*-terminal region of the core peptide (Feng et al. 2018). Grisemycin and some other type-A linaridins, such as the salinipeptins and cypemycin, contain multiple D-amino acids in their products. A recent study revealed that *grmL* from the grisemycin biosynthetic gene cluster with unknown function is indispensable for grisemycin production, potentially encoding a novel peptide epimerase (Shang et al. 2019, Xiao et al. 2022). In addition, the D-Asp in bottromycin A2 is formed in a non-enzymatic epimerization process following the formation of a thiazoline adjacent to an Asp in the precursor peptide. This spontaneous conversion is consistent with previous reports of epimerization of amino acids adjacent to carboxylated thiazolines (Liu and Thomas 2013, Crone et al. 2016). Genome mining has revealed the diversity and ubiquity of the family of RiPP epimerases, providing a broad tool library for introducing D-amino acids into peptides, which may facilitate the engineering of lanthipeptides with improved pharmaceutical properties.

### Methylation

Besides proteins, DNA, and RNA, RiPPs also serve as substrates for methylation. Post-translational methylation of peptides is a widespread modification in biological systems, mediated by methyltransferases that catalyse the addition of methyl groups, primarily donated by S-adenosylmethionine (SAM). Peptides are methylated at a number of different sites, including, but not limited to, nitrogen-containing side chains of arginine and lysine, as well as the N-terminus and C-terminus of peptides. Linaridins are a small group within the RiPPs family. All characterized linaridins harbour a conserved *α*-*N*-dimethylation through the action of a locally encoded methyltransferase (Mo et al. 2017, Georgiou et al. 2020). Cypemycin, a typical linaridin, has a *N,N*-dimethyl-alanine introduced by the SAM-dependent methyltransferase CypM (see Table 1), which is essential for its bioactivity (Claesen and Bibb 2010). Similarly, *α*-*N*-dimethylation was also shown to be essential for the antibiotic activity of plantazolicin, a peptide from the thiazole/oxazole-modified microcin group of RiPPs, as de-methylated plantazolicin was not active against *Bacillus anthracis* (Molohon et al. 2011). These findings demonstrate the important role of methylation in the biological activity of RiPPs.

Genes encoding methyltransferases are frequently found in Class I lanthipeptide biosynthetic clusters (Acedo et al. 2019, Xue et al. 2022). A recently characterized O-methyltransferase, OlvS_A_, which is encoded in a Class I lanthipeptide gene cluster from *Streptomyces olivaceus* NRRL B-3009, catalyses the methylation of highly conserved aspartate residues to the corresponding methyl-ester in the cyclic substrate, which is spontaneously converted to succinimide, followed by non-enzymatic hydrolysis to generate a β-amino acid, i.e. isoaspartate in the precursor peptide OlvA. In *silico* analysis combined with experimental results revealed that OlvS_A_ (termed LanS_A_) represents a new family of *O*-methyltransferases distinct from protein *L*-isoaspartate (*D*-aspartate) *O*-methyltransferases (PIMTs). The *in vivo* and *in vitro* reconstitution of *O*-methyltransferase OlvSA activity not only revealed its leader peptide-independence and SAM-dependent mechanism, but also laid the groundwork for its potential development as a methyl ester-modification bioengineering tool (Acedo et al. 2019). Another SAM-dependent methyltransferase, annotated as LahS_B_, was discovered within a putative lanthipeptide biosynthetic gene cluster (*lah*), which also contains the unusual Class II lanthipeptide synthetases LahM1/M2. The activity of LahS_B_ was confirmed *in vivo* (with an *E. coli* expression system) and *in vitro*, showing that it methylates the carboxylate of the precursor LahA peptide. Its activity is independent of the leader peptide and shows a certain tolerance to the amino acid residues it methylates, potentially serving as a methyl ester-introducing tool with high substrate tolerance for lanthipeptide engineering. Although the bioactivity of the peptide LahA is uncertain and the importance of the C-terminal methylation is unclear, modification of the C-terminus of RiPPs is probably a protective strategy against the action of carboxypeptidases (Huo et al. 2020). The Class II lanthipeptides archalan β and archalan γ are the first reported lanthipeptides from archaea, both containing one *N*-terminal methylation, while the associated methyltransferase genes were identified in their BGCs. Mining of 7157 archaeal genomes revealed chemically diverse and highly variable peptide products, highlighting the potential of archaea as an important source of bioactive peptides as well as novel chemical structure-installing enzymes (Liang et al. 2022). Besides Classes I and II lanthipeptides, methylation also occurred in the Class III lanthipeptide andalusicin A on its N-terminus. More importantly, *α-N* dimethylation was shown to be indispensable for its antimicrobial activity (Grigoreva et al. 2021). Furthermore, in recent years, a novel class of lanthipeptides, Class V lanthipeptides, has been reported, which are characterized by the co-occurrence of lanthionine rings and *α*-*N*-dimethylation (Ortiz-López et al. 2020). For example, cacaoidin contains an unprecedented *N,N*-dimethyl-lanthionine (NMe_2_-Lan), which was modified by a putative *O*-methyltransferase homologue (but displays low sequence similarity to the *O*-methyltransferase LanS in Class I lanthipeptide BGCs). Other examples are the lexapeptides, which contain an *N,N*-dimethyl-phenylalanine installed by the methyltransferase LxmM, i.e. homologous to the cypemycin *α*-*N*-methyltransferase, and pristinin A3, which contains *N,N*-dimethyl-β-methyllanthionine (NMe_2_-MeLan) introduced by a putative carminomycin 4-*O*-methyltransferase (Kloosterman et al. 2020, Ortiz-López et al. 2020, Xu et al. 2020). Although the function and enzymology of *N*-terminal dimethylation remain unclear in Class V lanthipeptides, genome-mining efforts in Class V lanthipeptide BGCs and established heterologous production systems will guide future investigations on these new lanthipeptide methylations (Román-Hurtado et al. 2021). There are still many surprising discoveries reported of novel RiPP methyltransferases, such as the methyltransferase SinM encoded in the salinipeptin gene cluster, which is predicted to modify *N,N*-dimethylalanine (Me_2_Ala) in the precursor peptide (Shang et al. 2019), and KgrB, a founding member of a widespread superfamily of Fe-S-containing methyltransferases, identified in the enteropeptin BGC (Clark et al. 2021).

The prevalence of methyltransferases in RiPP BGCs and the importance of methylation on the bioactivity of RiPPs may trigger the development of methyltransferases as a useful tool for structure and activity optimization of lanthipeptides (Marsh et al. 2010, Zhang et al. 2015, Acedo et al. 2019, Grigoreva et al. 2021, Xue et al. 2022). The bioengineering potential of RiPP methyltransferases has been demonstrated in several studies. For example, cypemycin *a-N*-methyltransferase CypM, which shows moderate catalytic promiscuity, can methylate the Class I lanthipeptide nisin and the Class II lanthipeptide haloduracin, where methylated nisin shows higher activity than native nisin (Zhang and Van Der Donk 2012). The β-methylation, which occurs very rarely in RiPPs, is a common modification in polyketides (PKs) and non-ribosomal peptides (NRPs). Characterization of the β-methylation mechanism of RiPP bottromycins revealed the potential to structurally mimic PKs/NRPs by employing these novel radical SAM methyltransferases involved in bottromycin biosynthesis (Gomez-Escribano et al. 2012, Huo et al. 2012, Crone et al. 2016, Franz et al. 2021). The radical SAM methyltransferase domain in OphA (involved in Omphalotin A biosynthesis) showed the possibility to introduce N-methyl groups in non-native substrates, including the NRPS-derived cyclosporin A-like peptides (Van Der Velden et al. 2017). Taking all these findings into account, together with the fact that many methylated peptides in natural bioactive peptides exhibit drug-like properties, methylation is a promising tool in lanthipeptide engineering to optimize the activity, stability, and bioavailability of peptide drugs.

### Halogenation

Halogenation is catalysed via flavin adenine dinucleotide (FADH_2_)-dependent or non-heme-iron-dependent halogenases. These enzymes install halogen atoms (chlorine, bromine, iodine, or fluorine) into aromatic and aliphatic substrates activated for electrophilic attack, offering more substrate selectivity than haloperoxidases. Halogenated natural products have been described to occur both in NRPs and RiPPs. Halogens play a vital role in determining the biological activity of these secondary metabolites, as the removal or replacement of halogen from the NRP-antibiotics chloramphenicol and vancomycin, e.g. profoundly affected their activity and potency (Harris et al. 1985, Dinos et al. 2016, Wenski et al. 2022).

FADH2-dependent halogenases in RiPPs have been shown to catalyse tryptophan-5-chlorination during the biosynthesis of the lanthipeptide NAI-107 (Castiglione et al. 2008, Foulston and Bibb 2010). Unlike most FADH2-dependent tryptophan halogenases that halogenate free tryptophan, the MibH halogenase involved in NAI-107 biosynthesis acts exclusively on tryptophan in its cognate peptide substrate (see Table 1). In addition, MibH requires prior modifications to be installed on the NAI-107 peptide for halogenation of the tryptophan indole, and its high substrate specificity limits the potential of using MibH as a general peptide chlorinase (Ortega et al. 2017). Interestingly, another study reported that the addition of potassium bromide (KBr) to the growth medium of the producer strains readily resulted in the formation of brominated variants of NAI-107. Br can substitute for Cl in microbial metabolites, and this may relate to the higher reactivity of Br^−^ ions compared to Cl^−^ ions and is consistent with the mechanism used by halogenating enzymes. This result suggests that the Trp halogenases, such as MibH, are also capable of efficiently incorporating Br into peptides (Cruz et al. 2015). Recent work included the RiPP halogenase MibH as a query sequence to mine the marine sponge metagenome for halogenating enzymes, and thus a MibH homologue, named SrpI, was detected in the sponge-derived RiPP/proteusin (*srp*) gene cluster. SrpI catalyses tryptophan-6-bromination in the core peptide, and, unlike MibH, SrpI has a certain substrate tolerance and can brominate unmodified peptides, thus having good potential as a broad-spectrum peptide tryptophanyl brominase in biocatalytic applications (Nguyen et al. 2021). RiPP halogenases can achieve selectivities that are often challenging to accomplish using synthetic methodologies, although their substrate specificity limits the development of these enzymes as a general-purpose peptide modification biocatalyst in synthetic biology. Further genome mining and engineering efforts may drive the discovery of those RiPPs halogenases that are better suited for combinatorial applications on a broader series of substrates, opening up the opportunity for functional engineering of novel lanthipeptides (Neumann et al. 2008, Crowe et al. 2021).

### β-amino acid incorporation

β-amino acids are amino acids that have a one-carbon extension on the standard α-amino acid backbone. β-amino acid incorporation confers stability to the secondary structures of peptides and often improves their biological activity (Cabrele et al. 2014, Lee et al. 2017, Evans et al. 2020). Unlike NRPs that contain a wide range of β-amino acids, to date, RiPPs discovered containing β-amino acid residues are extremely rare. However, recent genome-guided discoveries indicate that ribosomal β-amino acid products are broadly distributed and can be biosynthesized within RiPP pathways (Scott et al. 2022, Wang et al. 2022). This can be exemplified by the discovery of the PlpX splicease, which is a radical SAM enzyme that functions together with its partner protein PlpY, catalysing an unusual splicing reaction that involves tyramine excision from the backbone and reconnection of the remaining atoms to generate an α-keto-β-amino residue, creating a β-amino acid-containing RiPP metabolite (Morinaka et al. 2018) (Table 1). Mutational analysis demonstrated the ‘XYG’ motif present at the splice site, which can direct several splicing events in one precursor. Thus, the PlpXY-mediated reaction can be used for site-specific introduction of multiple α-keto-β-amino acids into gene-encoded precursor peptides (Morinaka et al. 2018). Their application was extended to achieve incorporation of various α-keto-β-amino acid residues at either the *C*- or *N*-terminal or internal positions of proteins, highlighting their diverse applications in synthetic biology (Lakis et al. 2022). Subsequent bioinformatic analysis established that this splicing transformation broadly occurs across the bacterial kingdom of life. The β-amino acid-containing products include many previously unrecognized RiPPs, and β-residues confer potent protease inhibitory activities and expanded structural diversities to these peptides (Scott et al. 2022). Another example of β-amino acid installation was found in the Class I lanthipeptide OlvA, which we also mentioned previously to be involved in methylation. The radical SAM-dependent *O*-methyltransferase, OlvSA, catalyses the re-arrangement of a highly conserved aspartate to a β-amino acid, i.e. isoaspartate (Acedo et al. 2019). A recent study reported a new RiPP, named kintamdin, containing a rare β-enamino acid that does not fall into any of the known groups of RiPPs. The phosphotransferase KinD and the lyase KinC encoded in the *kin* gene cluster from *Streptomyces* sp. RK44 are processive in the phosphorylation and elimination of Ser-7 to a β-enamino acid, i.e. (Z) 3-amino-acrylic acid (Aaa), although the underlying mechanism of KinCD has not been determined yet (Wang et al. 2022). The RiPP-based β-amino acid installation synthases provide an enzymatic route to engineering various β-residues into peptides and proteins, allowing an expanded structural scope and improved stability of lanthipeptides or other RiPPs.

### Ornithine incorporation

Ornithine is a non-canonical amino acid, i.e. it is not genetically encoded but is produced in nature via deguanidination of arginine, which plays an important role in the urea cycle. Ornithine is common in many bioactive non-ribosomal peptides, such as the antibiotics daptomycin and gramicidin S, but is rarely found to be incorporated into ribosomal peptides. However, the recently identified OspR, a cyanobacterial arginase-like enzyme encoded in the silent *osp* gene cluster from the cyanobacterium *Kamptonema* sp. PCC 6506, can install two ornithines in the antiviral RiPP landornamide A (Bösch et al. 2020) (Table 1). Using OspR as the query sequence, more bacterial arginase homologues were identified from diverse RiPP families. The arginase activity of several representatives (KspR, PhaR, ChdR, CwbR, BlhR, PacR, and DeaR) was verified in *E. coli*, and a broad range of peptide sequences were used as substrates to assess their level of promiscuity. The results showed that ornithines could be introduced into a wide range of substrates, including some NRP mimics, indicating that these arginases are generally promiscuous. The high substrate tolerance of these maturases opens up the opportunity for the incorporation of ornithine by peptide bioengineering (Mordhorst et al. 2020). Further study of these ornithine-containing RiPPs and their gene clusters revealed a new class of RiPP-derived fatty-acylated lipopeptides, the selidamides (Hubrich et al. 2022). This new class is characterized by fatty acyl units attached to (hydroxy) ornithine or lysine side chains, which are catalysed by the modifying enzymes of the GCN5-related *N*-acetyltransferase (GNAT). Taking the biosynthesis of phaeornamide from *Pseudophaeobacter arcticus* DSM 23566 as an example, the precursor peptide PhaA is firstly modified by PhaR, which converts arginine to ornithine, followed by hydroxylation of the ornithine residue by PhaI, generating 4(S)-hydroxy-2(S)-ornithine, and subsequent acylation of (hydroxy) for fatty acid attachment, catalysed by the GNAT PhaN. These PhaRIN co-mediated modifications highlight the potential of engineering peptides with diverse, non-ribosomal-like features in a ribosomal way (Hubrich et al. 2022). Furthermore, a bioinformatics study discovered an unusual type of sactipeptide, termed enteropeptins, featuring *N*-methylornithine modification. An arginine residue is deguanidinated to ornithine by a predicted Mn-dependent arginase and then *N*-methylated by a Fe-S-dependent methyltransferase, resulting in the first reported instance of *N*-methylornithine in a RiPP (Clark et al. 2021). The discovery of ornithine-installation enzymes and other RiPPs enzymes that catalyse downstream modifications based on ornithine enriches the biocatalytic toolbox and permits an important expansion of the chemical diversity of peptides by bioengineering.

### Glycosylation

Glycosylation refers to the process of transferring single or multiple types of sugar donors to acceptor molecules to form glycosides, the products of which are called glycocins in the RiPPs family. Glycosylation generally occurs on the side chains of Cys, Thr, or Ser amino acid residues to form S-glycocins or O-glycocins in RiPPs. Compared with other PTM reactions, such as cyclization and lipidation, glycosylation is relatively rare in RiPPs. Until now, only a few glycocins have been identified and characterized. Several studies have shown that S-linked glycocins are more stable than O-linked glycocins, both chemically and biologically (Oman et al. 2011, Wang et al. 2014). There are a few S-glycosides formed with UDP-glucose as the natural sugar donor. Sublancin 168, produced by *B. subtilis* 168, is the first reported S-linked glycocin containing a β-linked glucose moiety attached to the thiol of Cys22 (Oman et al. 2011, Wang and Van Der Donk 2011, Biswas et al. 2017, Ren et al. 2018). The peptide pallidocin also includes a glucose on Cys22, which is catalysed by PalS (Table 1), i.e. encoded by an essential gene in the pallidocin biosynthetic gene cluster from the thermophilic *Aeribacillus pallidus* eight strain (Kaunietis et al. 2019). Another S-glycosyltransferase, ThuS, is able to catalyse both S-glycosylation of the thiol of cysteine and O-glycosylation of the hydroxyl group of serine in Thurandacin B from *Bacillus thuringiensis serovar andalousiensis* BGSC 4AW1 (Wang et al. 2014). Although the specificities of these enzymes vary, they all have a high substrate tolerance. SunS and PalS can modify various analogues of natural substrates, and SunS is highly promiscuous with respect to its nucleotide-sugar donor and can accommodate different sugars such as UDP-α-D-N-acetylglucosamine (UDP-GlcNAc), UDP-α-D-galactose (UDP-Gal), guanosine diphosphate α-D-mannose (GDP-Man), and UDP-α-D-xylose (UDP-Xyl) to complete the glycosylation reaction (Oman et al. 2011). In addition, ThuS demonstrates a high tolerance with respect to both nucleotide sugars and peptide substrates. ThuS was able to glucosylate SunA despite the significant differences in the sequences of SunA and ThuA. However, SunS cannot modify ThuA, indicating that the tolerance of ThuS to peptides is higher than that of SunS (Wang et al. 2014). Structural and mechanistic investigations of ThuS and SunS reveal that they consist of an unusual glycosyltransferase Type A (GTA)-fold architecture and form a dimer to create an extended cavity to accommodate different peptide substrates (Fujinami et al. 2021). Overall, these S-glycosyltransferases offer promising potential to be used as a tool for the biosynthesis of glycosylated bioactive peptides. Furthermore, the S-glycoside glycocin F (GccF), i.e. secreted by *Lactobacillus plantarum* KW30, has an *N*-acetylhexosamine and not a UDP-glucose S-linked to Cys43, which is indispensable for its activity (Stepper et al. 2011, Drummond et al. 2021). Finally, the glycocin F homologue ASM1, produced by *Lactobacillus plantarum* A-1, differs from glycocin F in the C-terminal sequence of the peptide (Main et al. 2020).

In addition to S-glycocins, O-glycocins that attach a sugar moiety to Ser or Thr residues have also been reported, such as Enterocin F4-9 (Maky et al. 2015) and Enterocin 96 (Izquierdo et al. 2009). Enzymes derived from this family are amenable to peptide engineering as glycosyltransferases have a relatively broad and relaxed donor- and acceptor-substrate scope (Nagar and Rao 2017). The sugar donor of O-glycocins can also be attached to Tyr residues, like in the previously mentioned Class V lanthipeptide cacaoidin (Román-Hurtado et al. 2021). Furthermore, a glycosylated lanthipeptide, named NAI-112, has also been discovered. It contains a deoxyhexose modification, i.e. N-linked to a tryptophan residue, which is quite rare in the RiPPs family (Sheng et al. 2020). Glycosylation also occurs in lasso peptides, although their mechanism of action is unclear to date (Zyubko et al. 2019).

### Hydroxylation

Hydroxylation is a relatively abundant modification in both NRPs and RiPPs. Hydroxylation in the RiPPs family can occur either on aromatic moieties or on aliphatic amino acids to produce antibiotics with altered activity.

Thiopeptides represent an important RiPP class equipped with varied and remarkable bioactivities (Arnison et al. 2013). The family of thiopeptides typically features a characteristic macrocyclic core, which is composed of a six-membered, nitrogenous thiazole and multiple oxazoles and dehydroamino acids (Arnison et al. 2013, Zhang and Liu 2013). Some thiopeptides contain hydroxyl groups that are introduced by cytochrome P450 hydroxylases (Liu and Thomas 2013, Tocchetti et al. 2013, Zheng et al. 2016). Thiopeptide GE2270 contains a hydroxyl group located at the β-position of the amino acid phenylalanine, which is introduced by the cytochrome P450 monooxygenase PtbO, i.e. encoded in the gene cluster of the GE2270 producer *Planobispora rosea* (Tocchetti et al. 2013). In addition, the Ile10 of thiostrepton is di-hydroxylated, which is catalysed by TsrR, another cytochrome P450 enzyme (Zheng et al. 2016). Furthermore, two different types of hydroxyl groups exist in nosiheptide; one is attached to the γ-position of Glu6, which is a typical sp^3^ carbon. Another is put at the Pyr3 position, which is a rare sp^2^ carbon atom. These two modifications are performed by NosB and NosC, respectively, which belong to the P450 monooxygenases (Liu and Thomas 2013).

Hydroxylation is also reported in a family of lasso peptides, which feature a unique lariat-knot architecture. The first identified hydroxylated lasso peptide was Canucin A, which was discovered by activation of its silent biosynthetic gene cluster in *Streptomyces canus*. Further studies on the biosynthetic pathway of Canucin A showed that CanE, an a-ketoglutarate/iron (II)-dependent hydroxylase, could install the hydroxyl group at the β-carbon of the *C*-terminal aspartate residue on the precursor peptide, which occurs prior to macrocyclization. In addition, CanE could also modify Asn and Glu effectively but not Ala and Ser, which indicates that CanE has a certain substrate specificity, i.e. it is promising as part of a toolkit for the combinatorial biosynthesis of lasso peptides (Zhang and Seyedsayamdost 2020).

The β-hydroxylation of Asn can also be found in polytheonamides, which are among the first known members of the proteusin family of RiPPs. Polytheonamides represent the most heavily post-translationally modified biomolecules derived from amino acids described to date. Seven enzymes are responsible for installing ∼50 PTMs distributed across 49 residues, including epimerization, dehydration, methylation, and hydroxylation, among others. Co-expression studies confirmed that PoyI regio-selectively hydroxylates Val24, Val32, and Asn38. The V31 L and N38H of PoyA point mutants were tolerated by PoyI, while V31H and N38Q were not (Freeman et al. 2017). The recognition motifs of PoyI need to be further studied to enable the use of these enzymes as hydroxylation tools in synthetic biology.

Modification of Asn by hydroxylation also occurs in lanthipeptides, which generally contain lanthionine (Lan) and/or methyllanthionine (MeLan) rings. Duramycin consists of 19 amino acids and includes one Lan and two MeLans, an unusual lysinoalanine (Lal) bridge, and an erythro-3-hydroxy-L-aspartic acid. The modification of hydroxy-Asp is installed by an α-ketoglutarate/iron (II)-dependent hydroxylase, DurX (Table 1). A glycine residue next to the Asp residue is necessary for DurX activity, which was determined by mutational analysis of the DurA precursor peptide (Zimmermann et al. 1993, Huo et al. 2017, An et al. 2018). Further studies on these peptides and their derivatives prove that hydroxylation is important for the bioactivity of peptides (Ökesli et al. 2011, Huo et al. 2017). Hydroxylation in other amino acids than Asn can also be found in lanthipeptides. The di-hydroxylation of a pro residue by a cytochrome P450 enzyme in the biosynthesis of the lanthipeptide NAI-107 has been demonstrated (Maffioli et al. 2014).

The hydroxylation modification is also present in the dikaritins, like ustiloxin B (Ye et al. 2016) and asperipin-2a (Ye et al. 2019), although the mechanism of hydroxylation has not been elucidated to date. Although Asn-hydroxylation enzymes were found in different classes of RiPPs, the enzyme recognition motifs were different. Further studies on specific enzyme motifs are needed to use these enzymes for synthetic biology.

### Prenylation

Prenylation modification can increase the diversity of the (modified) peptide structure by adding carbon chains of different lengths at various positions of the peptide, thereby endowing the peptide with increased metabolic stability, enhanced membrane interactions, better peptide bioavailability, and improved pharmacokinetic and pharmacodynamic properties (Zhang and Bulaj 2012). Due to the significant impact on the properties of peptides following lipidation, lipidation has proven to be an effective tool in peptide engineering. There are some naturally occurring lipidated peptides with excellent antibacterial activities in NRPs, such as polymyxin B, which is active against Gram-negative bacteria (Velkov et al. 2010), and daptomycin, which kills Gram-positive bacteria (Baltz 2009). Recently, an increasing number of lipid modifications on peptides have also been found in RiPPs, in which prenyltransferases (PTases) from cyanobacteria play an important role.

The prenyltransferases from cyanobacteria (PTases) usually perform forward or reverse prenylation (5-carbon) or geranylation (10-carbon) reactions on Ser, Thr, or Tyr to produce various cyanobactins. In-depth studies performing structural, molecular dynamics, and biochemical characterization of prenyltransferases from cyanobacteria show that different enzymes have different specificities for their donors and acceptors. For instance, LynF (Table 1), PagF, PirF, and SphF can all accept tyrosine but recognize different donors (McIntosh et al. 2011, Hao et al. 2016, Martins et al. 2018, Morita et al. 2018). LynF, PagF, and SphF utilize 5-carbon moieties as donors, while PirF prefers a 10-membered carbon chain for the geranylation reaction. For some enzymes, even when recognizing the same donor and acceptor, the orientation of the prenyl group sometimes also varies. PagF and SphF carry out forward prenylation, while LynF performs reverse prenylation. The reverse O-prenylated Tyr then undergoes a spontaneous Claisen rearrangement to yield a forward C-prenylated product. Prenylation on Trp residues has also been discovered, where usually prenylation is performed at the *N*-terminus of the peptide, like for AcyF (Dalponte et al. 2018), or at the C-3 carbon of the indole ring, like for KgpF (Ishida et al. 1997). In addition to modifying the amino terminus of some linear cyanobactins by, for instance, AgeMTPT and MusF1/2, N-forward mono- or bis-prenylation also occurs on arginine, catalysed by AgcF (Phan et al. 2021). Recently, a unique and bifunctional prenyltransferase, LimF, originating from *Limnothrix* sp. CACIAM 69d, has been reported that catalyses histidine-C-geranylation and Tyr-O-geranylation. Interestingly, it can act on various non-natural substrates (Zhang et al. 2022). In fact, the prenyltransferases isolated and identified from cyanobacteria display a wide substrate scope. In addition to modifying their own natural cyclic peptide substrates, LynF, PagF, and PirF could also modify linear peptides and even single amino acids. As these enzymes have been discovered rather recently, there are not many examples of the introduction of prenylation into lanthipeptides, but based on the observed relaxed substrate specificity of these enzymes, the engineering of PTases to produce new-to-nature lanthipeptides will be an ongoing and important trend in the near future.

In addition, longer aliphatic chains can be introduced by the prenyltransferase ComQ involved in the biosynthesis of the *Bacillus* quorum-sensing pheromone ComX. ComQ introduces geranylation or farnesylation of a 15-membered carbon chain at the C-3 position of a Trp. The ComQ_natto_ from *B. subtilis* subsp. *natto* can accept a wide range of *N*- and/or *C*-terminally truncated analogues, even a single Trp. This demonstrates that ComQ_natto_ has a broad substrate tolerance, which opens up the potential of ComQ as a new tool for the introduction of long carbon chains in lanthipeptide engineering (Sugita et al. 2018).

### Acylation

Acylation can introduce an acyl or an acetyl group onto a peptide, which is common in NRPs but not as well represented in RiPPs. At present, only a few peptides with acetylation modification derived from RiPPs have been identified, mainly in lanthipeptides (Ozaki et al. 2017, Wiebach et al. 2018, 2020, Hubrich et al. 2022) and lasso peptides (Zong et al. 2018).

Acylation in RiPPs mainly occurs at the N-terminus of a peptide. In goadsporin, after removal of the leader peptide, the N-terminal amino group was acetylated in the presence of acetyl-CoA by GodH (Table 1), which is a GNAT domain-containing acetyltransferase (Ozaki et al. 2017). Acylation at the N-terminus of the peptide can also be followed by other biosynthetic pathways, like NRPs or PKS. Microvionin and Nocavionin contain a bis-methylated guanidino fatty acid attached to the acylated N-terminus of the peptide, which is derived from fatty acid or polyketide biosynthesis (Wiebach et al. 2018, 2020). The Piel group has recently reported the selidamides, which form a new family of ribosomally derived, fatty-acylated lipopeptides. The fatty acyl moieties are attached to the side chain of lysine or (hydroxy)-ornithine. The acylation reaction was catalysed by maturases of the GCN5-related N-acetyltransferase superfamily (Hubrich et al. 2022).

Acylation also occurs naturally in lasso peptides. Albusnodin is the first confirmed lasso peptide where there is experimental demonstration of an acetylation modification, which is a prerequisite for the biosynthesis of this kind of lasso peptide (Zong et al. 2018).

For acetylation, compared to using acetyl-CoA as a donor, the enzyme involved in lipolanthine synthesis has obvious advantages. In addition to accepting diverse donors, it can also directly modify peptide side chains rather than being limited to the N-terminus of the peptide, which is of great significance for increasing the diversity of peptides. This makes it possible to generate more active peptides with different acyl chain lengths *in vivo*. At the same time, more genetic and biochemical characterization is required to obtain a more comprehensive and deeper understanding of the related acetyltransferases, which are certainly promising tools for synthetic biology.

### Epoxidation

Epoxidation modification involves the conversion of a double bond to a three-membered ring, which is close to an equilateral triangle. Epoxides are usually highly reactive because of their high ring tension and polarized carbon-oxygen bonds (Fretland and Omiecinski 2000, Tang 2007, Amacher 2012, Morisseau 2013). The introduction of an epoxide group into peptides usually makes them easy targets for ring opening by nucleophilic groups such as an amine (-NH_2_) or thiol moieties (-HS-), which are frequently occurring in peptides (Brotzel and Mayr 2007). Since there are many proteins and reactive amine groups in biological systems, the new scaffolds typically undergo further enzymatic modification to generate mature and stable peptides. The cytochromes P450 can act on aromatic or double bonds of peptides to install epoxidation modifications in peptides (Lamb et al. 2013, Rydberg et al. 2014).

Epoxide modification occurs in a variety of natural products, including NRPs as well as RiPPs. In NRPs, cyclomarin A and cyclomarin B are equipped with the structural characteristics of epoxidation. This modification is performed by a cytochrome P450 enzyme named CymV (Schultz et al. 2008). Similarly, three-membered rings are also present in the structural features of RiPP-derived peptides. For instance, during the biosynthesis of thiostrepton, the cytochrome P450 enzyme TsrI carries out epoxidation of the quinaldic acid moiety of thiostrepton (Table 1). Epoxidation is imperative for subsequent modification steps to produce mature thiostrepton (Tocchetti et al. 2013, Zheng et al. 2016).

Due to the extremely high tension and reactivity of epoxides, further modifications are generally performed *in vivo* to produce more stable products. But even so, epoxides display important activities, especially anti-cancer activity, which deserves further investigation.

## Combinatorial engineering of various modifications into lanthipeptides

### Natural combinations of PTMs in lanthipeptides

**Figure 3.**
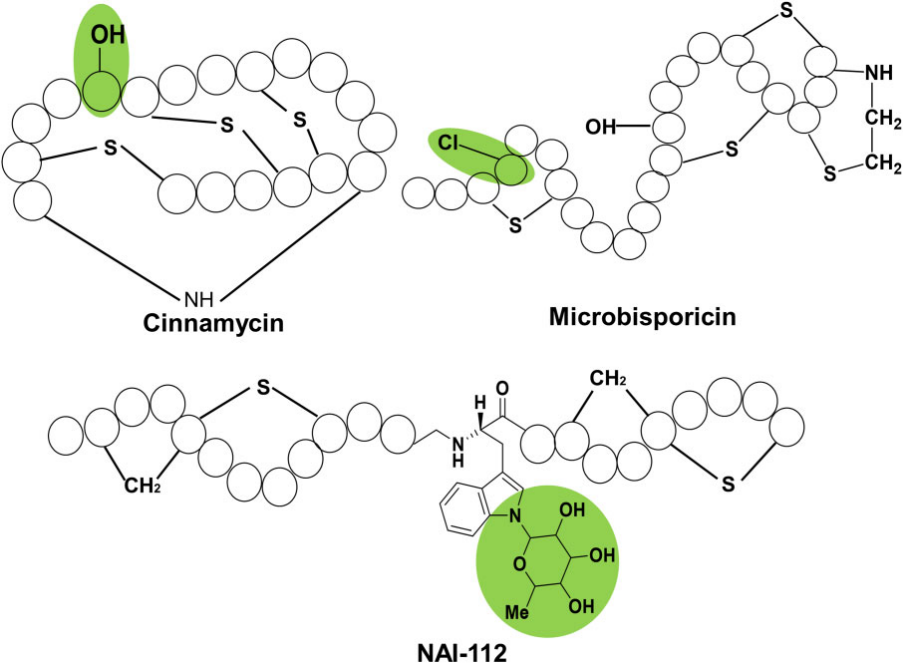
Natural combinations of diverse modifications in lanthipeptides. Cinnamycin and duramycin contain a hydroxyl group, which is essential for their antimicrobial activity. This group is marked in green in the Cinnamycin chemical structure; Lanthipeptide microbisporicin (NAI-107) contains a 5-chlorotryptophan (5-Cl-Trp) motif, which is shown in green in the corresponding structure; NAI-112, a glycosylated Class III lanthipeptide, contains a rare deoxyhexose modification N-linked to a tryptophan residue. This structural feature is highlighted in green.

In addition to the primary modification that forms the thioether rings, some lanthipeptides also contain various secondary modifications that introduce specific functional groups, and these diverse secondary modifications have a great impact on activity or stability or are necessary for the lanthipeptide synthesis or structure itself. Examples are shown in Fig. 3, where the hydroxylation of Asp15 of cinnamycin or duramycin is essential for their antimicrobial activity, as the hydroxyl group can form a hydrogen bond with the ammonium group of ethanolamine, which is part of the target lysophosphatidyl-ethanolamine (Hosoda et al. 1996, Ökesli et al. 2011, Huo et al. 2017). Furthermore, NAI-112 is a glycosylated Class III lanthipeptide produced by an *Actinoplanes* sp. strain with potent bioactivity against nociceptive pain, while weak antimicrobial activities are also displayed. It contains two labionin/methyllabionin motifs, and it also contains a rare deoxyhexose modification N-linked to a tryptophan residue, which is catalysed by the tailoring enzyme AplG (Sheng et al. 2020, Tocchetti et al. 2021). Another lanthipeptide, microbisporicin, contains 5-chlorotryptophan (5-Cl-Trp), which is formed by a halogenase named MibH. The halogenation modification is important for the activity of microbisporicin, because the 5-Cl-Trp residue is directly next to the presumed binding site of the N-acetylmuramic acid moiety (Cruz et al. 2015, Maffioli et al. 2016, Ortega et al. 2017). The combination of these natural Lan or MeLan with various tailoring modifications in lanthipeptides reflects the possibility of combining different modifications that confer diverse biological activities into one peptide, which is an inspirational motivation for creating new-to-nature molecules with specific and desired biological activities. In addition, enzymes with a high substrate tolerance, such as DurX, present in lanthipeptide biosynthetic pathways also create unprecedented possibilities for the introduction of diverse ‘foreign’ modifications into lanthipeptides. As these tailoring modifications also occur in other RiPPs families, it is likely that some of these enzymes may have been cross-combined at the genetic level. These notions all indicate the feasibility of combinatorial biology research with lanthipeptides as the starting point for engineering multiple modifications into lanthipeptides.

### Artificial combinations of PTMs into lanthipeptides

As RiPPs are synthesized by ribosomes, the primary sequence of the peptide can be changed by site-directed mutagenesis, which requires a certain substrate flexibility of the PTM enzymes of RiPPs. Several studies have shown the feasibility of this strategy. A large number of mutants of various lantibiotic precursor peptides can be produced by site-directed mutagenesis employing the biosynthetic mechanism in a heterologous or homologous host (Kuipers 1996, Cortés et al. 2009, Cooper et al. 2010). Both lacticin 3147 and mersacidin were investigated by full alanine scanning (Cotter et al. 2006, Appleyard et al. 2009). Random mutagenesis is also an approach with a high potential to generate vast genetically encoded libraries of natural-like lanthipeptides containing substantial structural diversity. For example, a precursor gene-encoded library of 10^6^ lanthipeptides has been generated *in vivo* by employing the promiscuous lanthipeptide synthetase ProcM, which led to the identification of an HIV p6 protein-human TSG101 protein interaction inhibitor library screening (Yang et al. 2018). Furthermore, Schmitt et al. used a large library of a variety of ring elements from 11 different lantibiotics to screen for improved activity with a novel nano-Fleming technology based on micro-alginate beads with a fluorescent producer target cells, and the protease NisP. In this way, they isolated >20 new-to-nature peptides with improved bioactivity (Schmitt et al. 2019). In addition, the *in vivo* activity of NisC allows for the cyclization of a wide array of unrelated and designed peptides that were fused to the nisin leader peptide (Kluskens et al. 2005, Rink et al. 2005, 2007). These *in vivo* studies have proven the remarkable promiscuous nature of lantibiotic biosynthetic enzymes. Lantibiotic biosynthetic enzymes can also install dehydro-amino acids or thioether rings in a large variety of non-native peptides attached to the native leader peptides. For example, the lanthipeptide synthetase LctM can not only modify lacticin 481 mutants, but is also able to modify other unrelated peptides containing thioether rings (Chatterjee et al. 2006, Levengood and van der Donk 2008). These studies highlight the promiscuity of these enzymes, although not all lantibiotic biosynthetic enzymes exhibit natural substrate promiscuity, which imposes limitations on installing lanthionines on non-natural substrates. However, lanthipeptide synthetases can be engineered to display different substrate promiscuities. For instance, a dehydratase mutant library of NisB with 10^5^ variants was generated via error-prone PCR. Subsequent high-throughput screening (HTS) based on cell surface display of the peptide products revealed a NisB variant that showed substrate flexibility against non-natural substrates (Zhao et al. 2020). Although mutagenesis of core sequences and protein engineering of PTM enzymes can increase the diversity of peptide structures, the structural variety that can be generated by such means is still limited.

As many modification enzymes in RiPPs recognize a leader or part of the leader to modify core peptides, diverse modifications from different biosynthetic pathways within the same family or across family boundaries can be combined into various core peptides by redesigning the leader peptide. In this way, several RiPP machineries have been combined to generate new and novel natural peptide mimics.

Nisin is one of the most studied lanthipeptides and is widely used as a natural food preservative. The nisin synthesis machinery was used to modify and secrete a putative two-component lantibiotic of *Streptococcus pneumoniae*, which was achieved by genetically fusing the sequences of spr1765 (pneA1) and spr1766 (pneA2) to the nisin leader-encoding sequence. The resulting RiPP harbours multiple dehydrated serine and/or threonine residues and (methyl) lanthionines. Both modified peptides displayed antimicrobial activity against *Micrococcus flavus* (Majchrzykiewicz et al. 2010). Chimeric leader peptides enable the combination of modifications introduced by RiPP maturases from unrelated pathways, given that the chimeric leader generated contains the corresponding recognition sequence (RS) for multiple modification enzymes. In this way, a chimeric leader containing recognition sequences for NisB/C and the thiazoline-forming cyclodehydratase HcaD/F enabled the creation of a new-to-nature thiazoline-lanthipeptide Class I hybrid. When the recognition sequence of the NisB/C leader sequence was swapped with the recognition sequence of ProcM, thiazoline-lanthipeptide Class II hybrid molecules could be generated. Furthermore, a thiazoline-sactipeptide hybrid could also be produced by this approach (Burkhart et al. 2017). Thus, the chimeric leader peptide strategy holds vast potential for combinatorial new-to-nature peptide generation.

**Figure 4.**
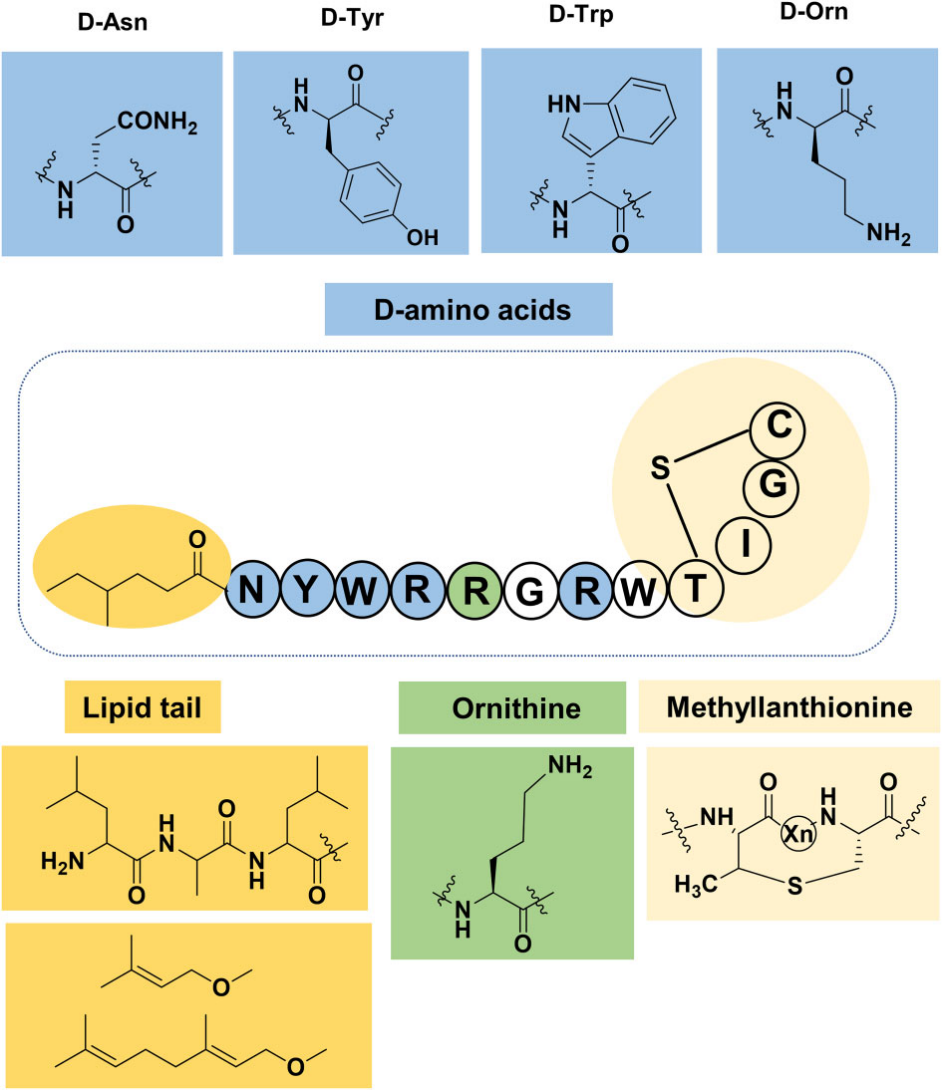
Schematic diagram of further modifications and combinations introduced into a brevicidine mimic. The implementation of mimicking mainly includes four parts. The fatty acid chains can be mimicked by using hydrophobic amino acids, or they can be catalysed by enzymes. The mimic of a lactone ring by a lanthionine and the fatty acid mimicking by three hydrophobic AA residues have already been achieved (Zhao et al. 2020). Further modification work mainly focuses on the incorporation of D-amino acids, the non-canonical amino acid ornithine, and the mimicking of a fatty acid chain by enzymatic methods. Piel’s group has demonstrated the possibility of introducing D-amino acids and ornithines onto similar linear peptide sequences (Mordhorst et al. 2020).

Combinatorial RiPP biosynthesis may also lead to the generation of NRP mimics, as much of the chemical complexity present in NRPs is also found in RiPPs. Since NRPs are not directly gene-encoded and are synthesized through large, multi-modular enzymes, the generation of NRP variants is challenging. Therefore, in recent years, the possibility of making mimics of NRPs with increased biological activity or improved physical or chemical properties by RiPP biosynthetic pathways has attracted a lot of interest. The Kuipers group initiated an important step towards this goal. They described a strategy to synthesize NRPs, mimics of the recently discovered non-ribosomal antimicrobial peptide brevicidine, employing nisin biosynthetic enzymes (Fig. 2). In this work, the lactone moiety was replaced by a thioether ring, and the fatty acid chains of NRPs were mimicked by a few hydrophobic amino acids. The engineered mimics showed antimicrobial activity against *Xanthomonas campestris*, which demonstrated that the structural mimicking of NRP by RiPP biosynthesis is feasible and offers great opportunities for engineering a wide range of effective antibiotics (Zhao et al. 2020). In addition, studies by Piel’s group have shown that OspR was able to modify three Arg residues to the non-canonical ornithine in another linear brevicidine mimic. In addition, the epimerase OspD could introduce a D-amino acid in this mimic, although the position of the D-amino acid did not match the D-amino acid positions in natural brevicidine (Mordhorst et al. 2020). These results indicate that there are a variety of RiPP tools available that can potentially be combined to introduce NRP structural features into ribosomal peptides (Fig. 4).

## Screening new-to-nature lanthipeptides with different methods

As illustrated in the preceding sections, large genetically encoded libraries of lanthipeptide variants can be generated by mutagenesis, which calls for the development of novel methods for the identification of desirable variants. Great efforts have been made to develop HTS methods to select variants from large libraries against specific targets, which can then be prepared in large quantities for further structure and bioactivity characterization.

**Figure 5.**
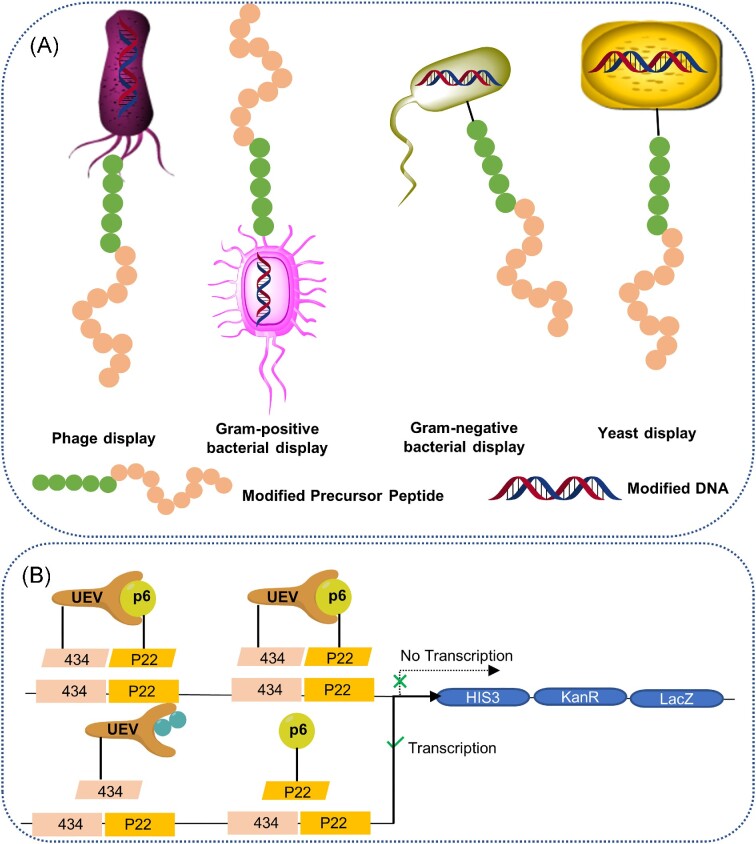
Schematic diagram of various screening methods. (A) Schematic diagram of phage, bacteria, and yeast surface display systems (Bosma et al. 2011, Urban et al. 2017, Hetrick et al. 2018). (B) Bacterial reverse two-hybrid technology for identifying a lanthipeptide inhibitor of the p6-UEV PPI (Yang et al. 2018).

Surface display technologies, including bacterial, yeast, and phage display technologies, represent an effective means of identifying peptides that bind to a cell, protein, or other molecules of interest. Different surface display technologies have been used in lanthipeptide screening, as shown in Fig. 5A. The first successful lanthipeptide surface display system was constructed in *L. lactis* and demonstrated to be effective in screening libraries with up to 10^9^ variants of lanthipeptides (Bosma et al. 2011). Other examples of successful selection of desirable lanthipeptide variants have been shown for the Class II lanthipeptide lacticin 481, for which an analogue with novel binding activity for αvβ3 integrin was screened from a 6 × 10^5^ library by yeast surface display combined with fluorescence activated cell sorting (FACS) as potential ligands for tumour imaging. (Hetrick et al. 2018). In the same study, a nisin variant with a different lipid II binding mode than the native nisin was identified from a library encoding 1.2 × 10^6^ variants by N-terminal phage display (Hetrick et al. 2018). With the knowledge that C-terminal fusions should be advantageous for the study of protein-protein interactions requiring free carboxy-termini (Fuh and Sidhu 2000), a C-terminal phage display system was developed. Applying this system, artificial lanthipeptide ligands specific to urokinase plasminogen activator (uPA) and streptavidin were readily identified from large C-terminal display libraries (Urban et al. 2017). Not limited to lanthipeptides, the bacterial surface display technique was also successfully implemented for the screening of high-affinity ligands specific for the VEGFA binding site on neuropilin-1 from a 6 × 10^9^ member bacterial display library derived from the cyclotide Kalata B1 scaffold, and a C-to-N cyclized cyclotide variant was identified as a potent antagonist of neuropilin that inhibits endothelial cell migration (Getz et al. 2013).

In addition, a bacterial reverse two-hybrid technology was developed that allowed for the identification and characterization of protein-protein interactions, leading to the discovery of antiviral lanthipeptide variants that prevent the interaction between the HIV p6 protein and the UEV domain of the human TSG101 protein, an interaction i.e. required for the HIV viral budding process (Yang et al. 2018). One interaction inhibitor was screened out from a 10^6^-non-natural lanthipeptide library, which was constructed in *E. coli* using the substrate-tolerant lanthipeptide synthetase ProcM (Fig. 5B). Such libraries, containing substantial structural diversity, may be combined with other cell-based assays to identify lanthipeptides with new biological activities (Yang et al. 2018).

Furthermore, colony-based assays are widely utilized for HTS of natural product analogues and have been exploited in a recent study to screen Class II lanthipeptide haloduracin analogues by in-colony removal of leader peptides in *E. coli* (Si et al. 2018). The main design principle underlying this approach is cellular compartmentalization, where the post-translational modifications of precursor peptides are completed in the cytosol and the leader peptide removal is programmed by a protease located in the periplasmic space. Subsequently, autolysis of *E. coli* cells is induced, permitting extracellular release of the final product for biological activity screening and analysis. This method is suitable for HTS of RiPPs variants that are inactive in the presence of the attached leader peptide (Si et al. 2018).

**Figure 6.**
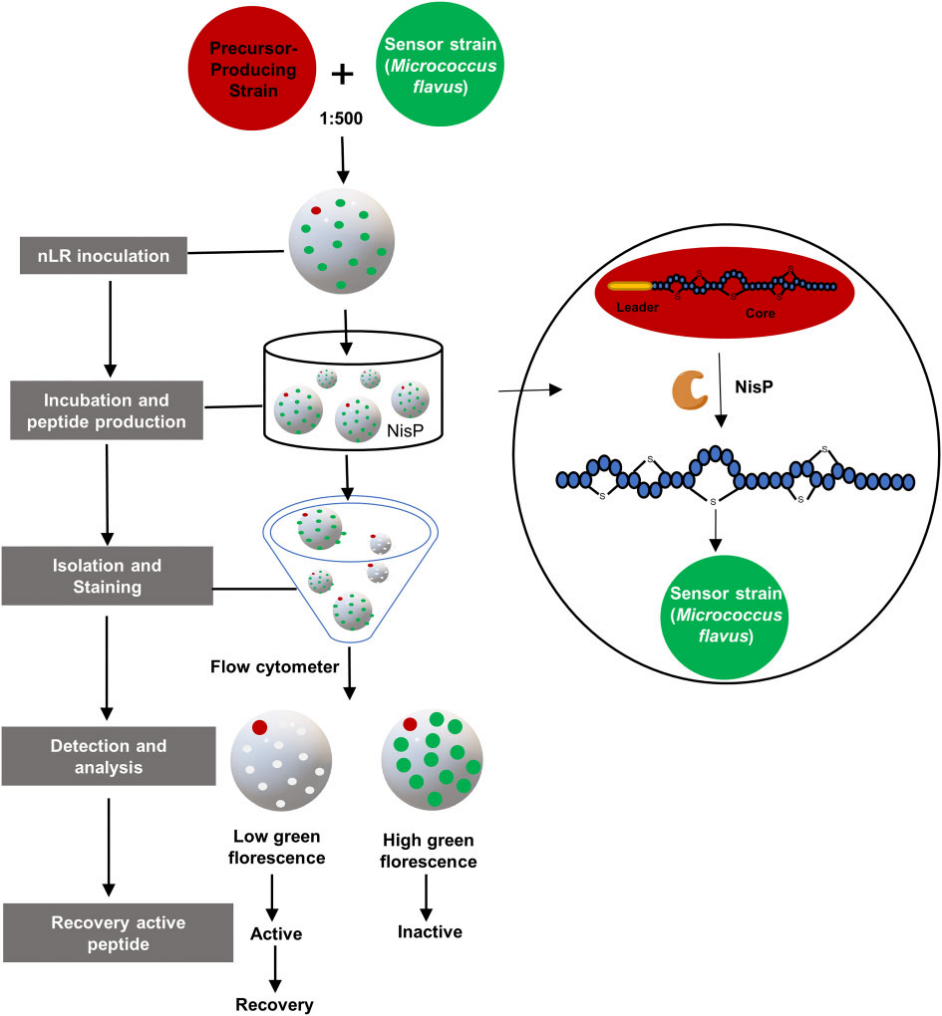
A schematic diagram for nanoFleming technology application in active peptide screening (Schmitt et al. 2019).

More recently, the nano-Fleming, a miniaturized and parallelized high-throughput inhibition assay, was developed to screen 6000 combinatorial lanthipeptide variants at the nanoliter (nL) scale. The fluorescently labelled peptide producer and sensor cells were encapsulated into nanoliter reactors (nLRs) for growth and peptide production. The nLRs with a small number of sensor cells were identified as containing potential producers of lanthipeptide variants, as shown in Fig. 6. With this combinatorial approach, a number of antimicrobial lanthipeptides that showed improved activity over wild-type peptides or were able to bypass resistance mechanisms were identified (Schmitt et al. 2019).

The studies of high-throughput sequencing methods in other RiPP families also provide concepts that can be extended to lanthipeptides. For example, mRNA display technology compatible with Flexizyme reprogramming allows the introduction of non-natural amino acids into the RiPP antibiotic pantocin, with the capacity to screen large and diverse libraries containing multiple simultaneous mutations (Fleming et al. 2020). Various screening methods that have been developed so far and that are expected to be developed in the future continuously foster the evolution of lanthipeptide engineering. Achieving the discovery of new-to-nature lanthipeptides with enhanced pharmacological properties and therapeutic possibilities from large *in vivo* lanthipeptide engineering libraries is a challenging but realistic goal.

## Conclusion and outlook

**Figure 7.**
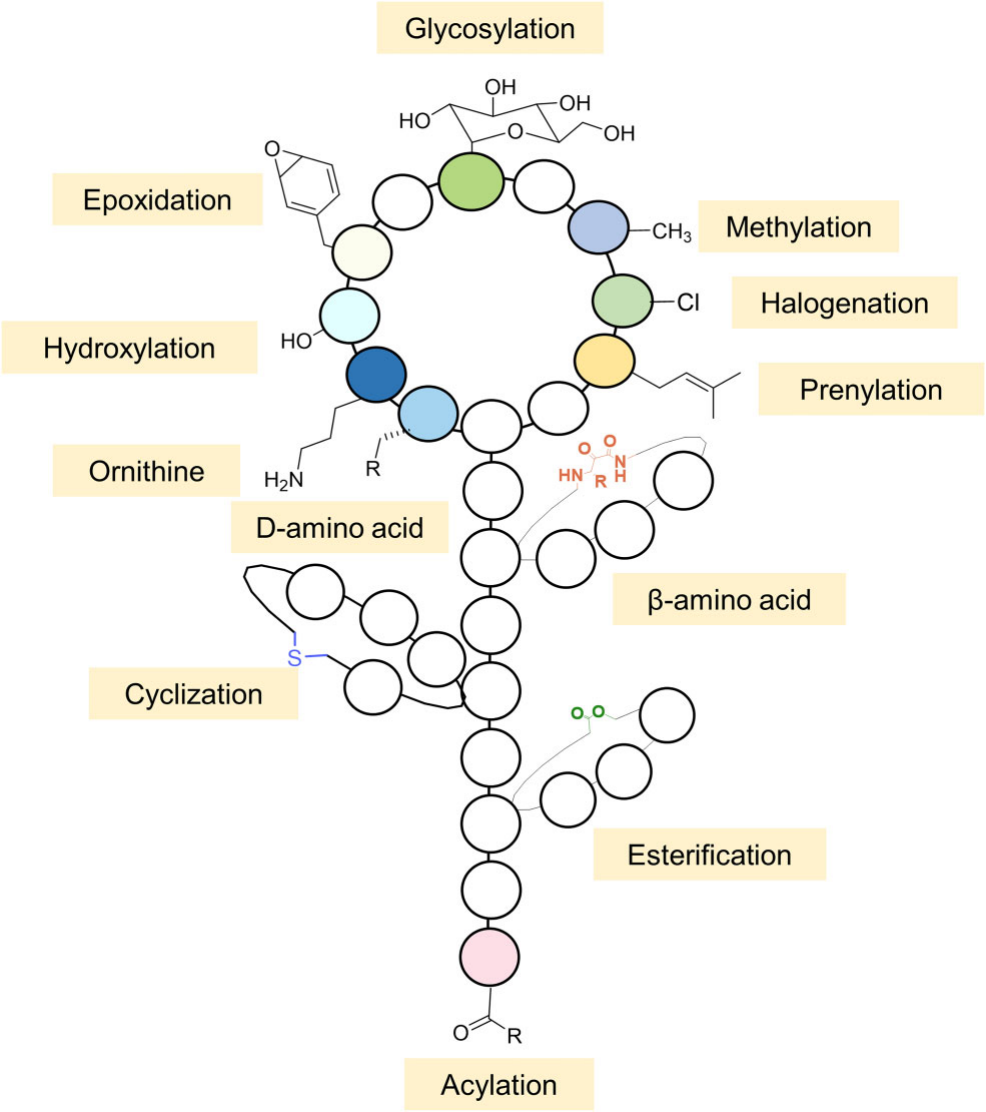
Artist’s ‘floral’ impression of combinations of diverse modifications into one peptide, putatively made possible by the use of the RiPPs enzyme.

The diverse PTM enzymes from RiPPs constitute a valuable biocatalytic toolbox that can be used for lanthipeptide and other RiPP engineering. Combinatorial application of these tools in lanthipeptide biosynthetic assembly lines allows for an expanded structural scope and enhanced or altered bioactivities of lanthipeptides, enabling the development of an invaluable resource for bioactive compounds and potential drugs. Several strategies for the combined use of these enzymatic tools have been proposed and successfully demonstrated in the above-mentioned studies. In addition, leader-independent enzymes, such as the dehydrogenase LanJ and the *O*-methyltransferase OlvsS_A_, are well suited for introducing additional RiPPs modifications into various core peptides. Moreover, enzymes with high substrate tolerance, such as the lanthipeptide synthetase SyncM, the prenyltransferase ComQ, and the peptide arginase OspR, are good candidates for combinational use to create non-natural peptides. However, the implementation of some RiPPs enzymes in combinatorial biosynthesis is still challenging; e.g. the halogenase MibH requires prior modifications to be installed on the precursor peptide for halogenation of the tryptophan indole, and its high substrate specificity limits the potential of using MibH as a general peptide chlorinase. Other examples include enzymes with unknown function and enzymology, such as the putative *O*-methyltransferase responsible for N-terminal dimethylation in cacaoidin, or enzymes with an unclear recognition motif, such as the Fe(II)-2-ketoglutarate-dependent enzyme PoyI responsible for hydroxylation in polytheonamides. Further genome mining and engineering efforts are required to discover homologues of those enzymes that are better suited for combinatorial applications on a broader series of substrates. Moreover, the development of new strategies, e.g. the leader-peptide-free strategy as used for the cyclodehydratase LynD, will further boost the field of lanthipeptide engineering. The engineering studies from different research groups could also be combined to accelerate the development of molecular engineering of new-to-nature bioactive peptides with desired structural properties and biological functions, and Fig. 7 depicts this blueprint. Considering the intensive ongoing efforts in RiPPs engineering, it can be expected that many new-to-nature modified peptides will be produced in the near future, providing a rich source for new putative antibiotics to be used in human therapies. Importantly, these studies should also be followed by strong efforts in determining their stability, toxicity, pharmacodynamics, pharmacokinetics, and administration in mammals.
